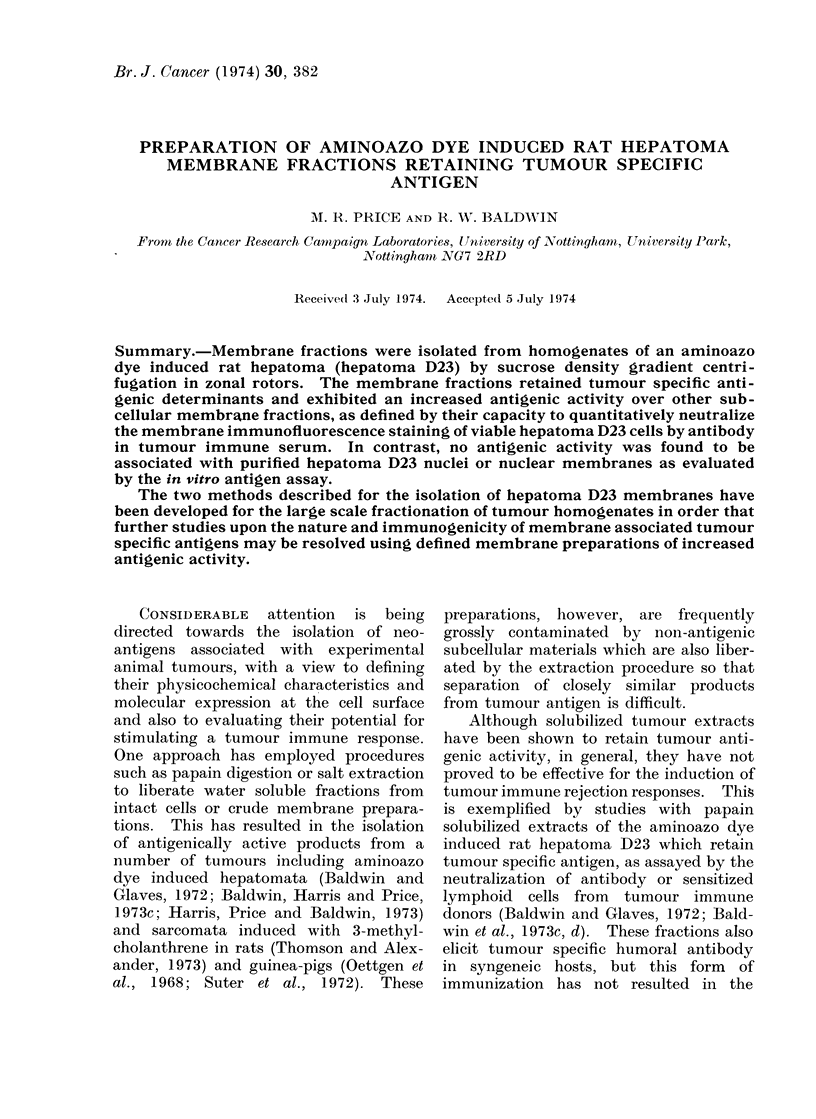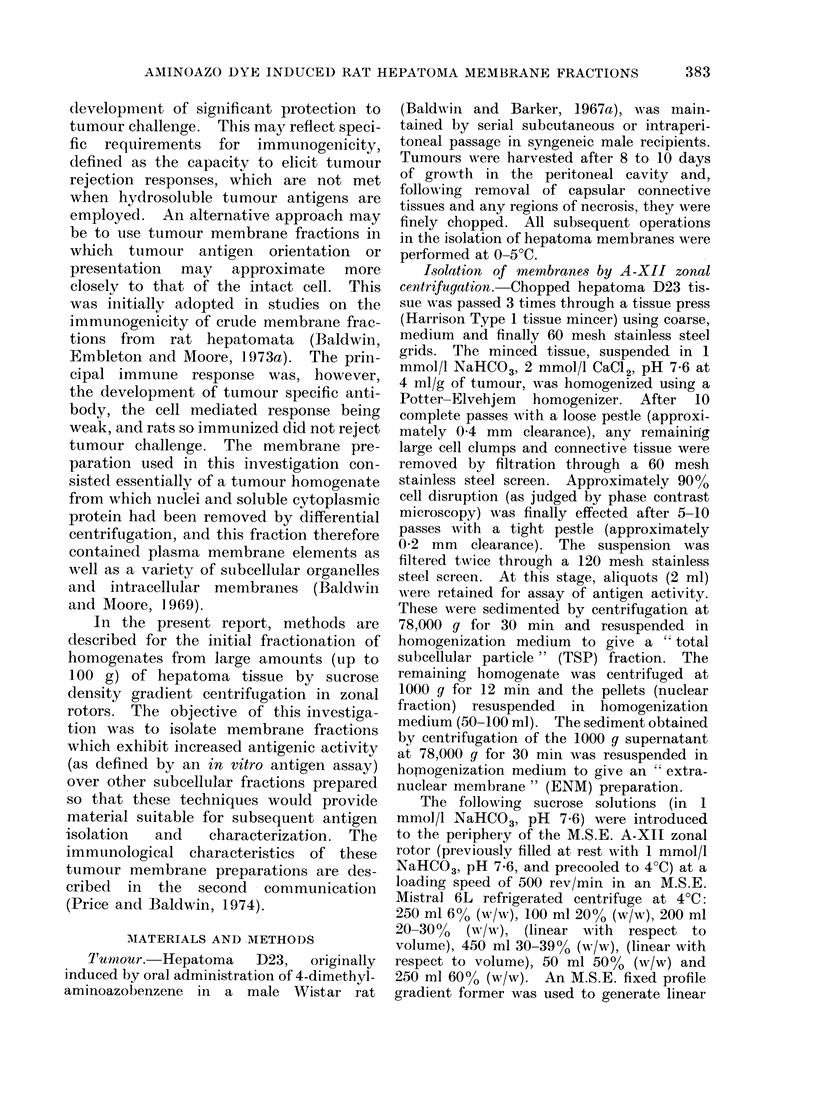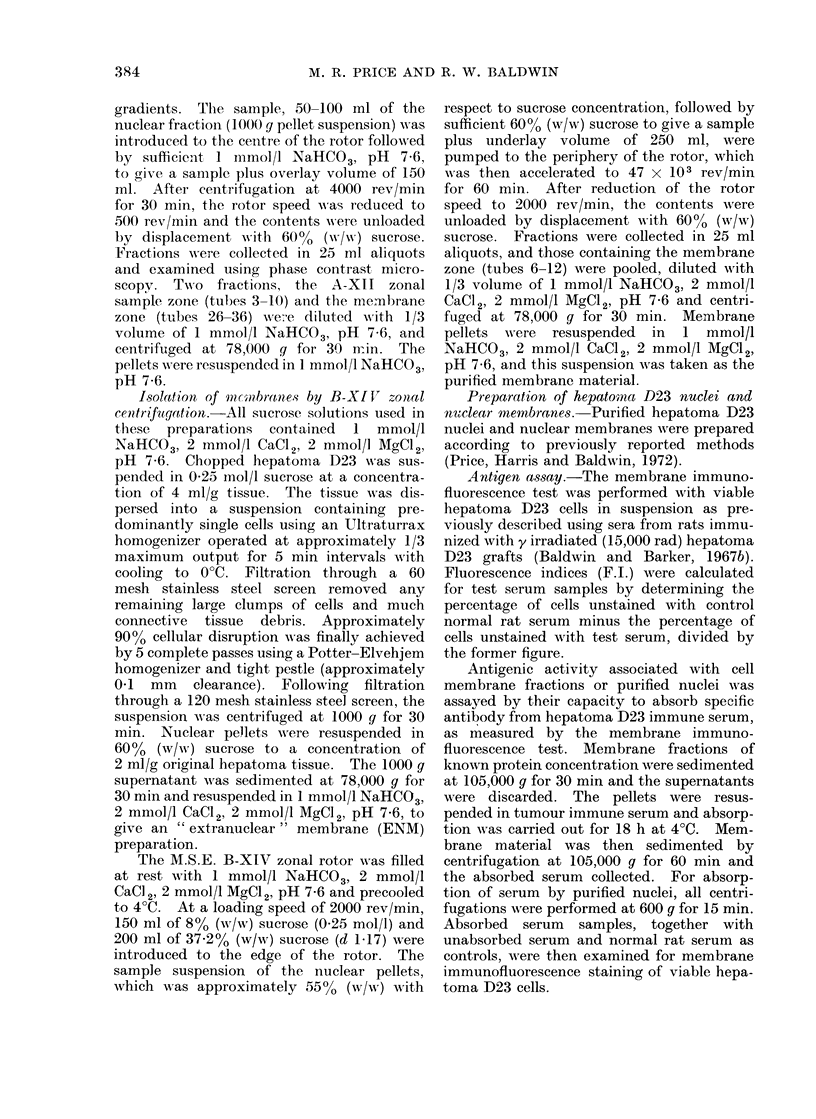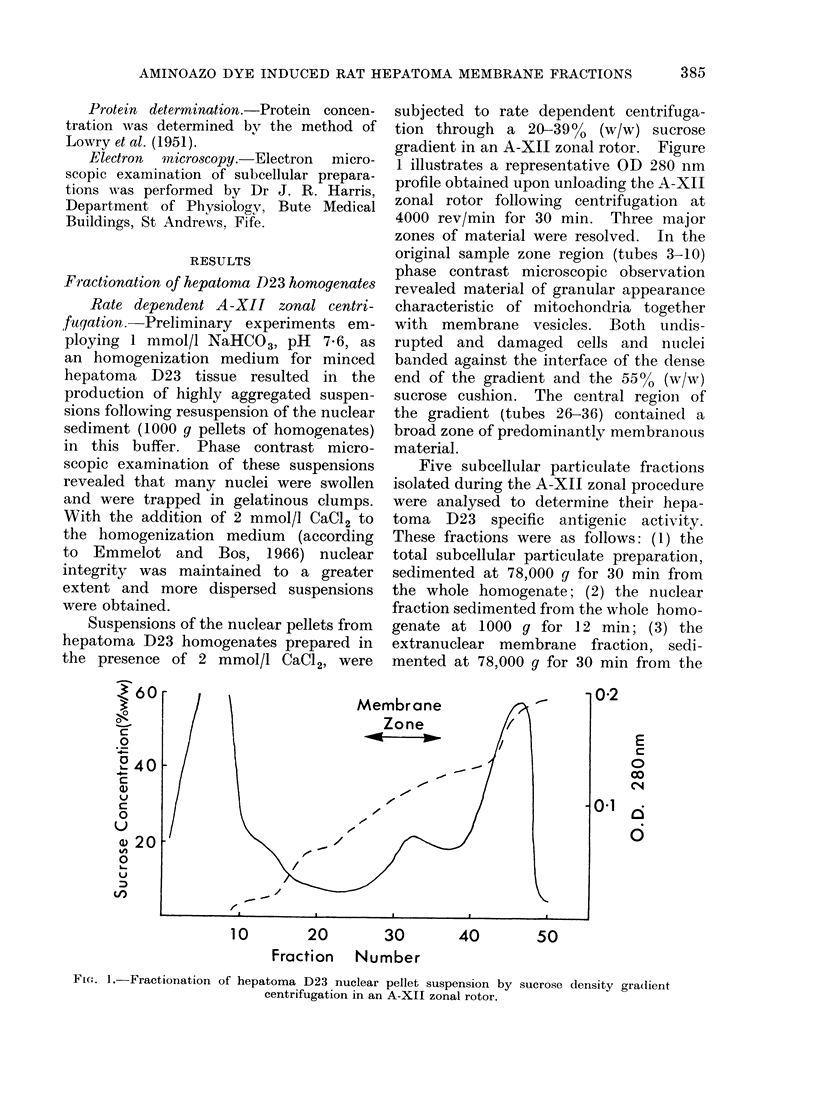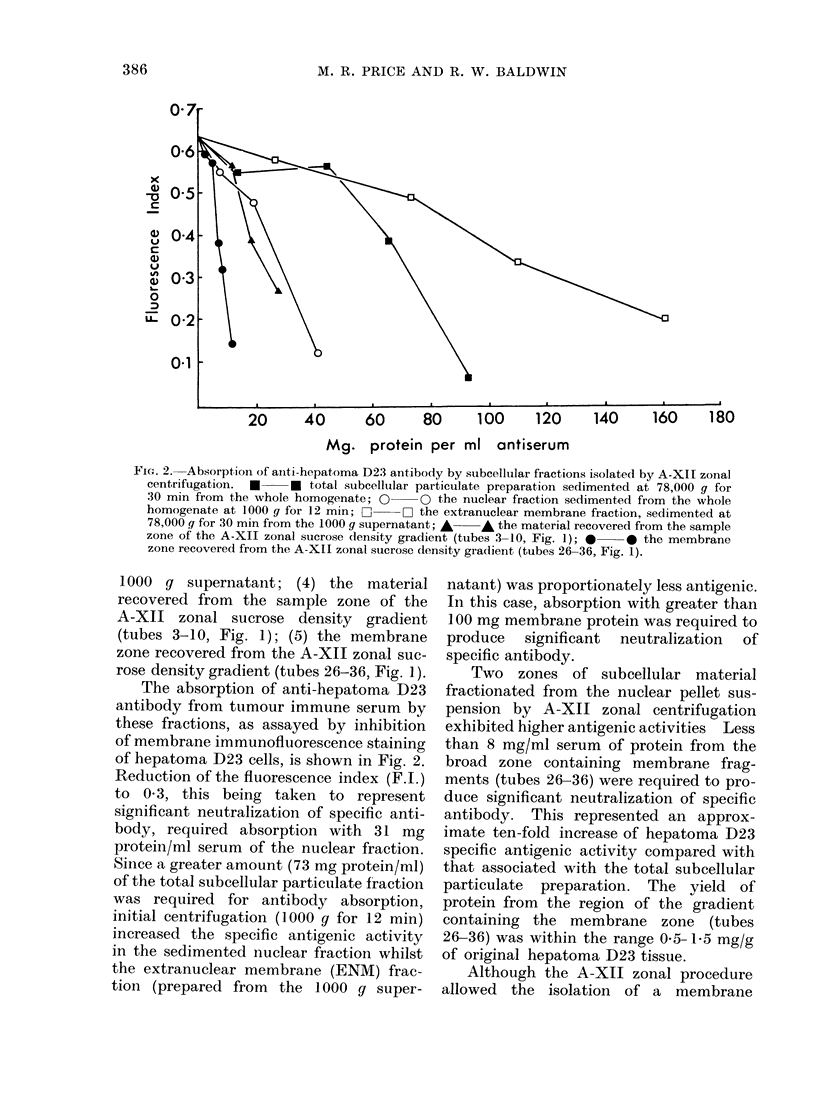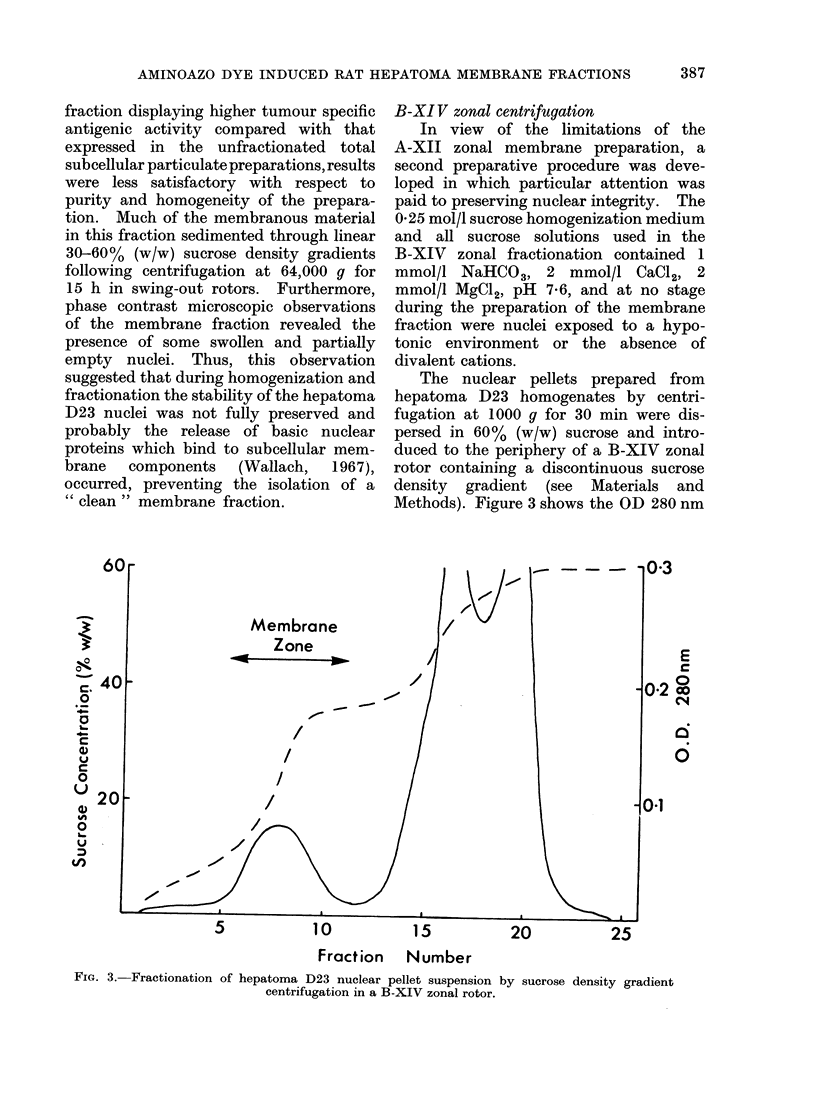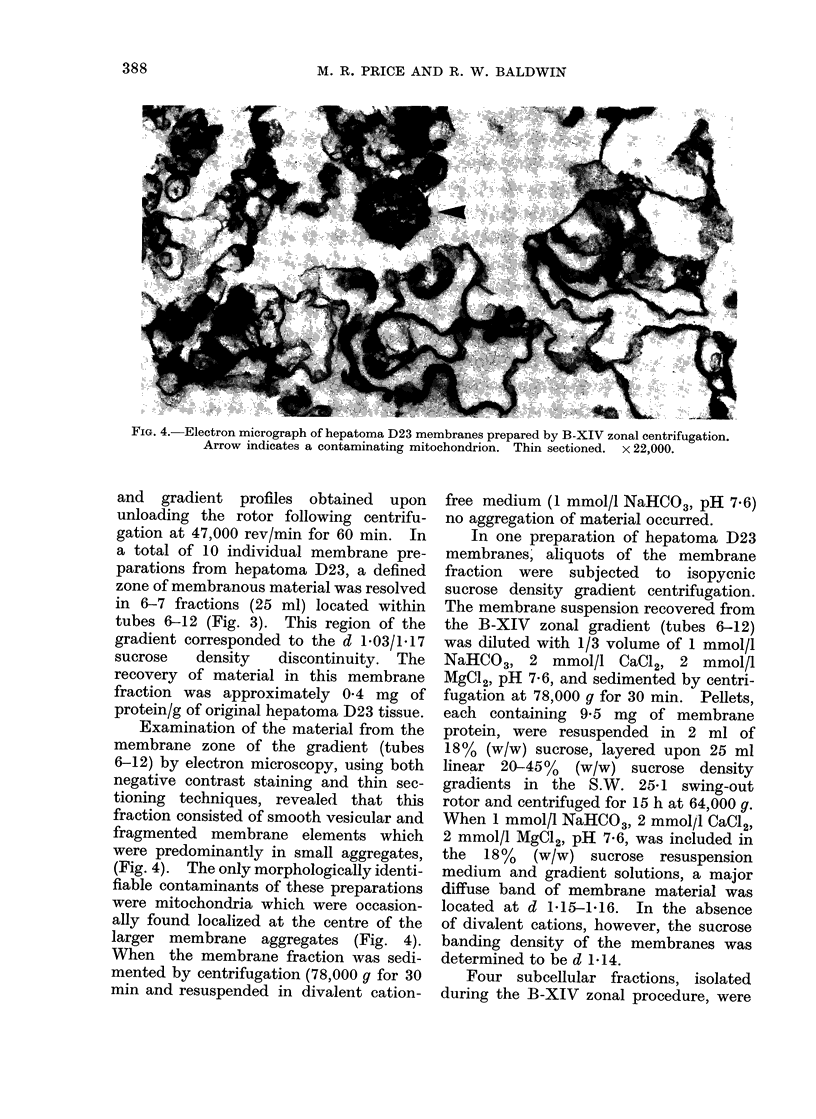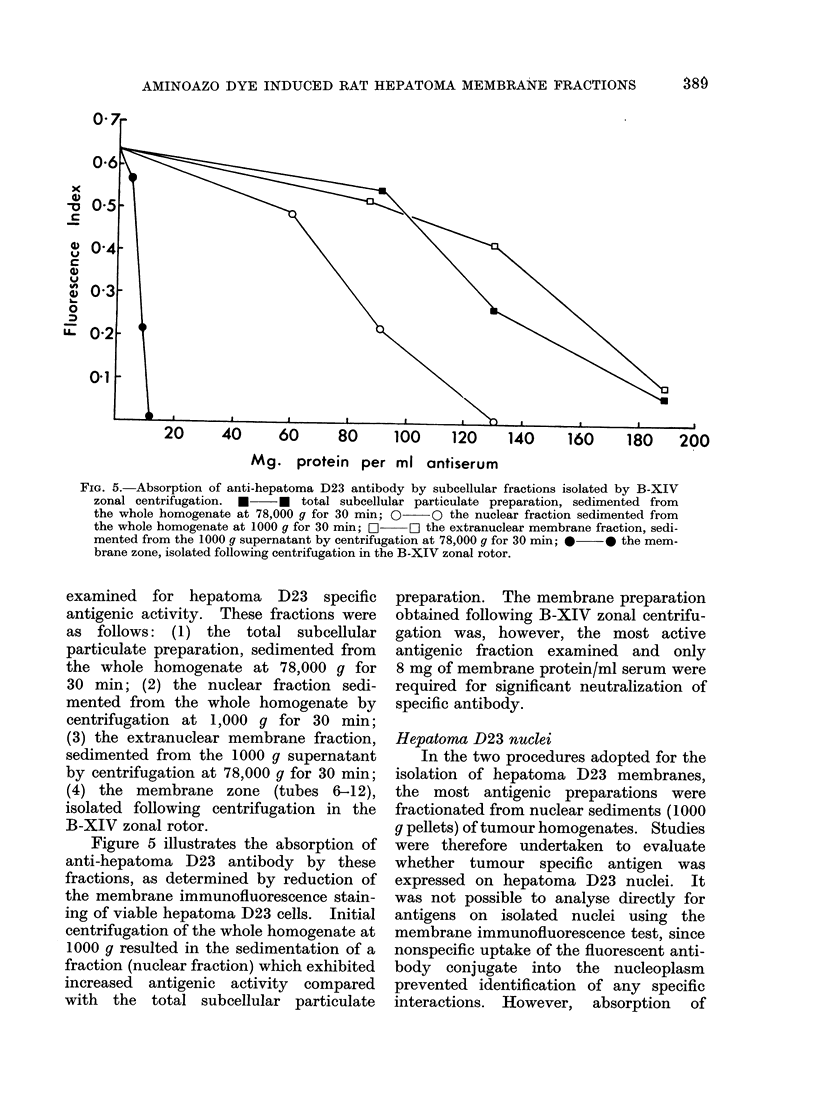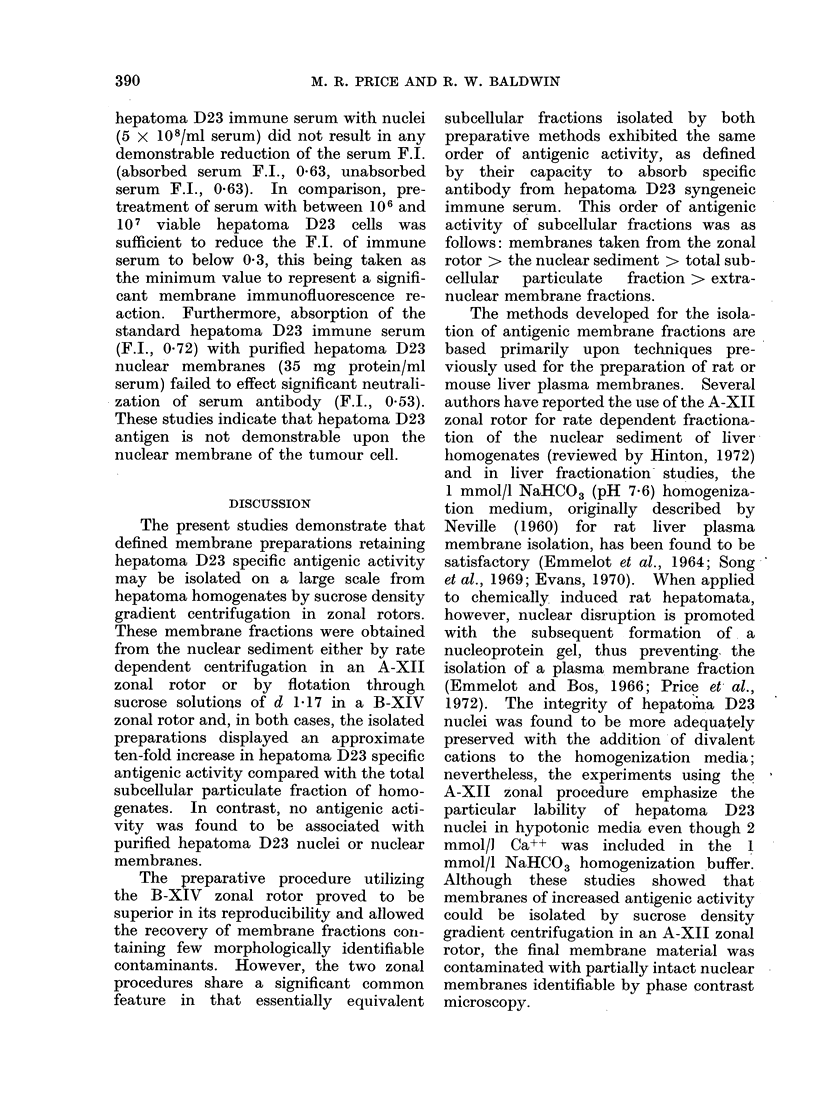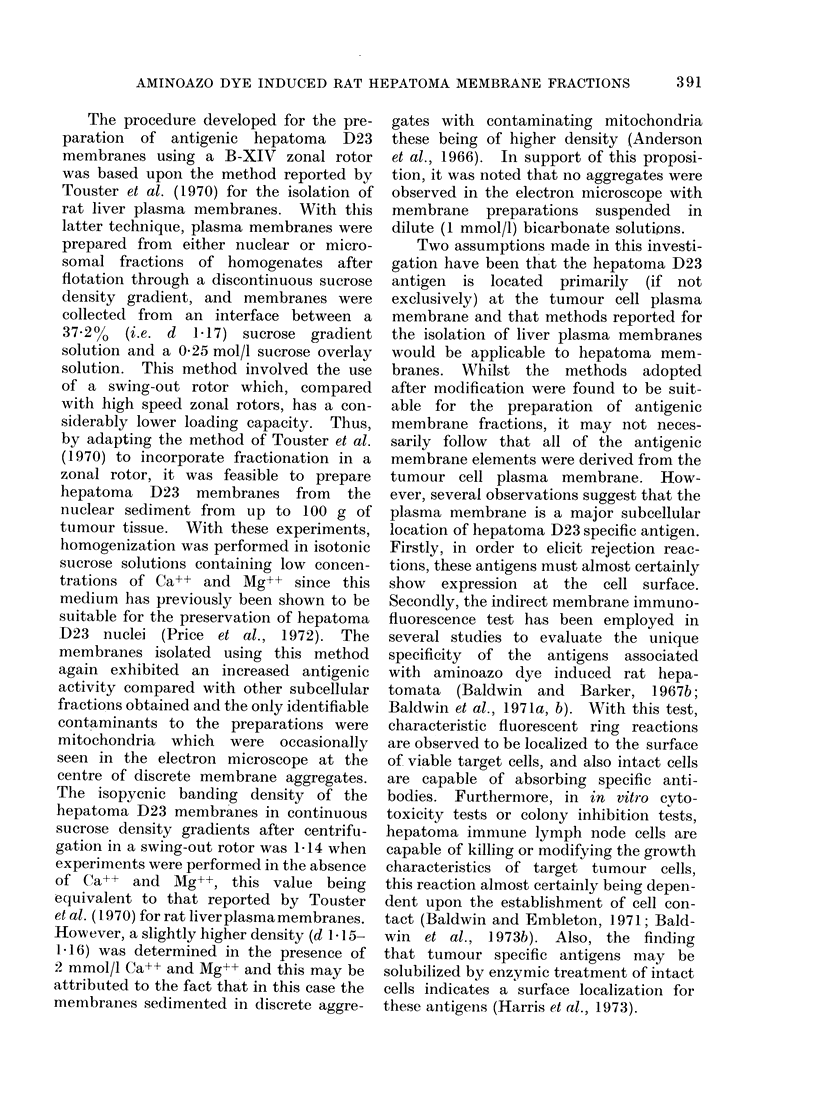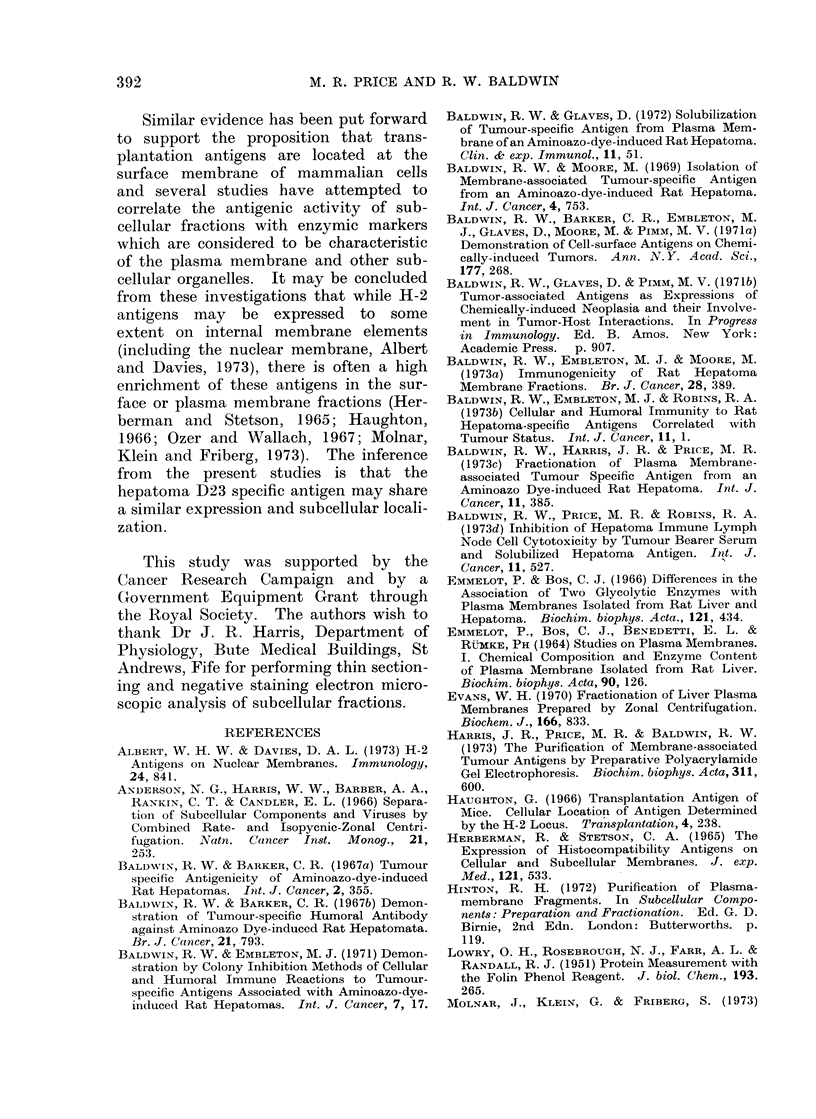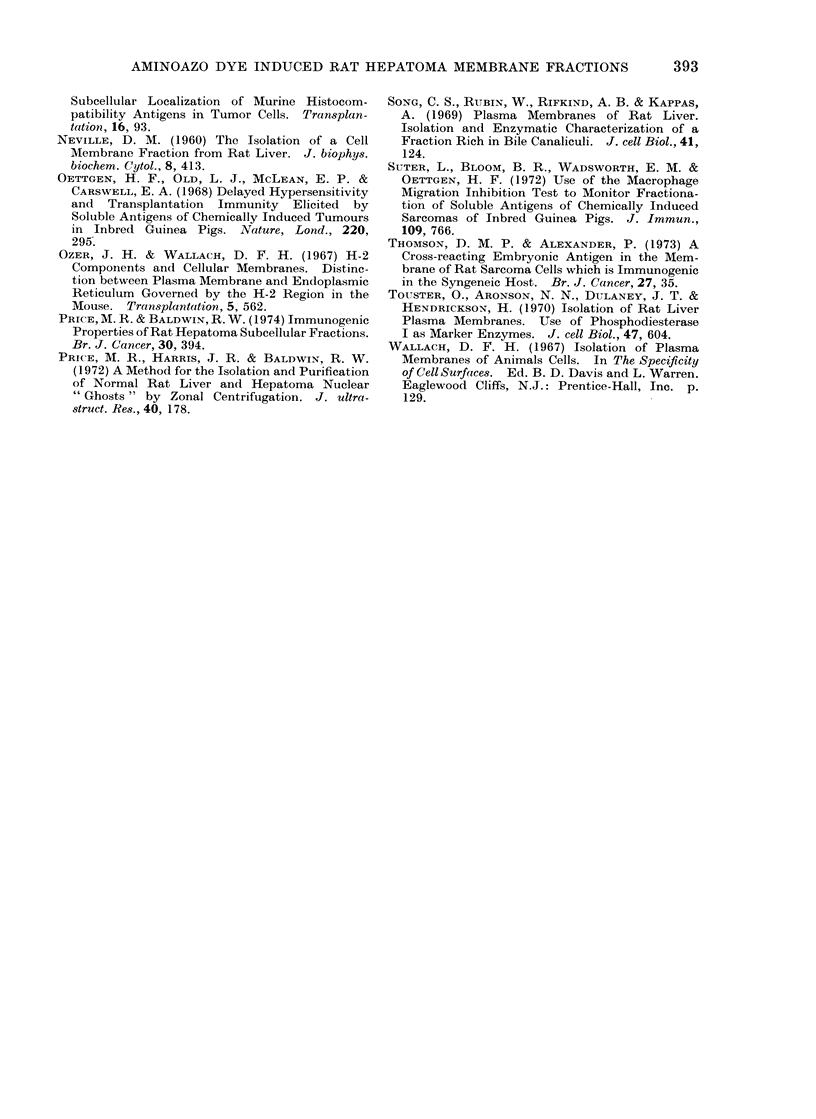# Preparation of Aminoazo Dye Induced Rat Hepatoma Membrane Fractions Retaining Tumour Specific Antigen

**DOI:** 10.1038/bjc.1974.212

**Published:** 1974-11

**Authors:** M. R. Price, R. W. Baldwin

## Abstract

**Images:**


*Br. J. Cancer* (1974) **30**, 382

### PREPARATION OF AMINOAZO DYE INDUCED RAT HEPATOMA MEMBRANE FRACTIONS RETAINING TUMOUR SPECIFIC ANTIGEN

M. R. PRICE AND R. W. BALDWIN

*From the Cancer Research Campaign Laboratories, University of Nottingham, University Park, Nottingham NG7 2RD*

Received 3 July 1974. Accepted 5 July 1974

**Summary.—Membrane fractions were isolated from homogenates of an aminoazo dye induced rat hepatoma (hepatoma D23) by sucrose density gradient centrifugation in zonal rotors. The membrane fractions retained tumour specific antigenic determinants and exhibited an increased antigenic activity over other subcellular membrane fractions, as defined by their capacity to quantitatively neutralize the membrane immunofluorescence staining of viable hepatoma D23 cells by antibody in tumour immune serum. In contrast, no antigenic activity was found to be associated with purified hepatoma D23 nuclei or nuclear membranes as evaluated by the *in vitro* antigen assay.**

**The two methods described for the isolation of hepatoma D23 membranes have been developed for the large scale fractionation of tumour homogenates in order that further studies upon the nature and immunogenicity of membrane associated tumour specific antigens may be resolved using defined membrane preparations of increased antigenic activity.**

CONSIDERABLE attention is being directed towards the isolation of neoantigens associated with experimental animal tumours, with a view to defining their physicochemical characteristics and molecular expression at the cell surface and also to evaluating their potential for stimulating a tumour immune response. One approach has employed procedures such as papain digestion or salt extraction to liberate water soluble fractions from intact cells or crude membrane preparations. This has resulted in the isolation of antigenically active products from a number of tumours including aminoazo dye induced hepatomata (Baldwin and Glaves, 1972; Baldwin, Harris and Price, 1973*c*; Harris, Price and Baldwin, 1973) and sarcomata induced with 3-methylcholanthrene in rats (Thomson and Alexander, 1973) and guinea-pigs (Oettgen *et al.*, 1968; Suter *et al.*, 1972). These preparations, however, are frequently grossly contaminated by non-antigenic subcellular materials which are also liberated by the extraction procedure so that separation of closely similar products from tumour antigen is difficult.

Although solubilized tumour extracts have been shown to retain tumour antigenic activity, in general, they have not proved to be effective for the induction of tumour immune rejection responses. This is exemplified by studies with papain solubilized extracts of the aminoazo dye induced rat hepatoma D23 which retain tumour specific antigen, as assayed by the neutralization of antibody or sensitized lymphoid cells from tumour immune donors (Baldwin and Glaves, 1972; Baldwin *et al.*, 1973*c, d*). These fractions also elicit tumour specific humoral antibody in syngeneic hosts, but this form of immunization has not resulted in the development of significant protection to tumour challenge. This may reflect specific requirements for immunogenicity, defined as the capacity to elicit tumour rejection responses, which are not met when hydrosoluble tumour antigens are employed. An alternative approach may be to use tumour membrane fractions in which tumour antigen orientation or presentation may approximate more closely to that of the intact cell. This was initially adopted in studies on the immunogenicity of crude membrane fractions from rat hepatomata (Baldwin, Embleton and Moore, 1973*a*). The principal immune response was, however, the development of tumour specific antibody, the cell mediated response being weak, and rats so immunized did not reject tumour challenge. The membrane preparation used in this investigation consisted essentially of a tumour homogenate from which nuclei and soluble cytoplasmic protein had been removed by differential centrifugation, and this fraction therefore contained plasma membrane elements as well as a variety of subcellular organelles and intracellular membranes (Baldwin and Moore, 1969).

In the present report, methods are described for the initial fractionation of homogenates from large amounts (up to 100 g) of hepatoma tissue by sucrose density gradient centrifugation in zonal rotors. The objective of this investigation was to isolate membrane fractions which exhibit increased antigenic activity (as defined by an *in vitro* antigen assay) over other subcellular fractions prepared so that these techniques would provide material suitable for subsequent antigen isolation and characterization. The immunological characteristics of these tumour membrane preparations are described in the second communication (Price and Baldwin, 1974).

### MATERIALS AND METHODS

*Tumour.*—Hepatoma D23, originally induced by oral administration of 4-dimethylaminoazobenzene in a male Wistar rat (Baldwin and Barker, 1967*a*), was maintained by serial subcutaneous or intraperitoneal passage in syngeneic male recipients. Tumours were harvested after 8 to 10 days of growth in the peritoneal cavity and, following removal of capsular connective tissues and any regions of necrosis, they were finely chopped. All subsequent operations in the isolation of hepatoma membranes were performed at 0–5°C.

*Isolation of membranes by A-XII zonal centrifugation.*—Chopped hepatoma D23 tissue was passed 3 times through a tissue press (Harrison Type 1 tissue mincer) using coarse, medium and finally 60 mesh stainless steel grids. The minced tissue, suspended in 1 mmol/l $NaHCO_3$, 2 mmol/l $CaCl_2$, pH 7·6 at 4 ml/g of tumour, was homogenized using a Potter–Elvehjem homogenizer. After 10 complete passes with a loose pestle (approximately 0·4 mm clearance), any remaining large cell clumps and connective tissue were removed by filtration through a 60 mesh stainless steel screen. Approximately 90% cell disruption (as judged by phase contrast microscopy) was finally effected after 5–10 passes with a tight pestle (approximately 0·2 mm clearance). The suspension was filtered twice through a 120 mesh stainless steel screen. At this stage, aliquots (2 ml) were retained for assay of antigen activity. These were sedimented by centrifugation at 78,000 *g* for 30 min and resuspended in homogenization medium to give a "total subcellular particle" (TSP) fraction. The remaining homogenate was centrifuged at 1000 *g* for 12 min and the pellets (nuclear fraction) resuspended in homogenization medium (50–100 ml). The sediment obtained by centrifugation of the 1000 *g* supernatant at 78,000 *g* for 30 min was resuspended in homogenization medium to give an "extranuclear membrane" (ENM) preparation.

The following sucrose solutions (in 1 mmol/l $NaHCO_3$, pH 7·6) were introduced to the periphery of the M.S.E. A-XII zonal rotor (previously filled at rest with 1 mmol/l $NaHCO_3$, pH 7·6, and precooled to 4°C) at a loading speed of 500 rev/min in an M.S.E. Mistral 6L refrigerated centrifuge at 4°C: 250 ml 6% (w/w), 100 ml 20% (w/w), 200 ml 20–30% (w/w), (linear with respect to volume), 450 ml 30–39% (w/w), (linear with respect to volume), 50 ml 50% (w/w) and 250 ml 60% (w/w). An M.S.E. fixed profile gradient former was used to generate linear gradients. The sample, 50–100 ml of the nuclear fraction (1000 *g* pellet suspension) was introduced to the centre of the rotor followed by sufficient 1 mmol/l $NaHCO_3$, pH 7·6, to give a sample plus overlay volume of 150 ml. After centrifugation at 4000 rev/min for 30 min, the rotor speed was reduced to 500 rev/min and the contents were unloaded by displacement with 60% (w/w) sucrose. Fractions were collected in 25 ml aliquots and examined using phase contrast microscopy. Two fractions, the A-XII zonal sample zone (tubes 3–10) and the membrane zone (tubes 26–36) were diluted with 1/3 volume of 1 mmol/l $NaHCO_3$, pH 7·6, and centrifuged at 78,000 *g* for 30 min. The pellets were resuspended in 1 mmol/l $NaHCO_3$, pH 7·6.

*Isolation of membranes by B-XIV zonal centrifugation.*—All sucrose solutions used in these preparations contained 1 mmol/l $NaHCO_3$, 2 mmol/l $CaCl_2$, 2 mmol/l $MgCl_2$, pH 7·6. Chopped hepatoma D23 was suspended in 0·25 mol/l sucrose at a concentration of 4 ml/g tissue. The tissue was dispersed into a suspension containing predominantly single cells using an Ultraturrax homogenizer operated at approximately 1/3 maximum output for 5 min intervals with cooling to 0°C. Filtration through a 60 mesh stainless steel screen removed any remaining large clumps of cells and much connective tissue debris. Approximately 90% cellular disruption was finally achieved by 5 complete passes using a Potter–Elvehjem homogenizer and tight pestle (approximately 0·1 mm clearance). Following filtration through a 120 mesh stainless steel screen, the suspension was centrifuged at 1000 *g* for 30 min. Nuclear pellets were resuspended in 60% (w/w) sucrose to a concentration of 2 ml/g original hepatoma tissue. The 1000 *g* supernatant was sedimented at 78,000 *g* for 30 min and resuspended in 1 mmol/l $NaHCO_3$, 2 mmol/l $CaCl_2$, 2 mmol/l $MgCl_2$, pH 7·6, to give an "extranuclear" membrane (ENM) preparation.

The M.S.E. B-XIV zonal rotor was filled at rest with 1 mmol/l $NaHCO_3$, 2 mmol/l $CaCl_2$, 2 mmol/l $MgCl_2$, pH 7·6 and precooled to 4°C. At a loading speed of 2000 rev/min, 150 ml of 8% (w/w) sucrose (0·25 mol/l) and 200 ml of 37·2% (w/w) sucrose (*d* 1·17) were introduced to the edge of the rotor. The sample suspension of the nuclear pellets, which was approximately 55% (w/w) with respect to sucrose concentration, followed by sufficient 60% (w/w) sucrose to give a sample plus underlay volume of 250 ml, were pumped to the periphery of the rotor, which was then accelerated to $47 \times 10^3$ rev/min for 60 min. After reduction of the rotor speed to 2000 rev/min, the contents were unloaded by displacement with 60% (w/w) sucrose. Fractions were collected in 25 ml aliquots, and those containing the membrane zone (tubes 6–12) were pooled, diluted with 1/3 volume of 1 mmol/l $NaHCO_3$, 2 mmol/l $CaCl_2$, 2 mmol/l $MgCl_2$, pH 7·6 and centrifuged at 78,000 *g* for 30 min. Membrane pellets were resuspended in 1 mmol/l $NaHCO_3$, 2 mmol/l $CaCl_2$, 2 mmol/l $MgCl_2$, pH 7·6, and this suspension was taken as the purified membrane material.

*Preparation of hepatoma D23 nuclei and nuclear membranes.*—Purified hepatoma D23 nuclei and nuclear membranes were prepared according to previously reported methods (Price, Harris and Baldwin, 1972).

*Antigen assay.*—The membrane immunofluorescence test was performed with viable hepatoma D23 cells in suspension as previously described using sera from rats immunized with $\gamma$ irradiated (15,000 rad) hepatoma D23 grafts (Baldwin and Barker, 1967*b*). Fluorescence indices (F.I.) were calculated for test serum samples by determining the percentage of cells unstained with control normal rat serum minus the percentage of cells unstained with test serum, divided by the former figure.

Antigenic activity associated with cell membrane fractions or purified nuclei was assayed by their capacity to absorb specific antibody from hepatoma D23 immune serum, as measured by the membrane immunofluorescence test. Membrane fractions of known protein concentration were sedimented at 105,000 *g* for 30 min and the supernatants were discarded. The pellets were resuspended in tumour immune serum and absorption was carried out for 18 h at 4°C. Membrane material was then sedimented by centrifugation at 105,000 *g* for 60 min and the absorbed serum collected. For absorption of serum by purified nuclei, all centrifugations were performed at 600 *g* for 15 min. Absorbed serum samples, together with unabsorbed serum and normal rat serum as controls, were then examined for membrane immunofluorescence staining of viable hepatoma D23 cells.

*Protein determination.*—Protein concentration was determined by the method of Lowry *et al.* (1951).

*Electron microscopy.*—Electron microscopic examination of subcellular preparations was performed by Dr J. R. Harris, Department of Physiology, Bute Medical Buildings, St Andrews, Fife.

### RESULTS

### *Fractionation of hepatoma D23 homogenates*

*Rate dependent A-XII zonal centrifugation.*—Preliminary experiments employing 1 mmol/l $NaHCO_3$, pH 7·6, as an homogenization medium for minced hepatoma D23 tissue resulted in the production of highly aggregated suspensions following resuspension of the nuclear sediment (1000 *g* pellets of homogenates) in this buffer. Phase contrast microscopic examination of these suspensions revealed that many nuclei were swollen and were trapped in gelatinous clumps. With the addition of 2 mmol/l $CaCl_2$ to the homogenization medium (according to Emmelot and Bos, 1966) nuclear integrity was maintained to a greater extent and more dispersed suspensions were obtained.

Suspensions of the nuclear pellets from hepatoma D23 homogenates prepared in the presence of 2 mmol/l $CaCl_2$, were subjected to rate dependent centrifugation through a 20–39% (w/w) sucrose gradient in an A-XII zonal rotor. Figure 1 illustrates a representative OD 280 nm profile obtained upon unloading the A-XII zonal rotor following centrifugation at 4000 rev/min for 30 min. Three major zones of material were resolved. In the original sample zone region (tubes 3–10) phase contrast microscopic observation revealed material of granular appearance characteristic of mitochondria together with membrane vesicles. Both undisrupted and damaged cells and nuclei banded against the interface of the dense end of the gradient and the 55% (w/w) sucrose cushion. The central region of the gradient (tubes 26–36) contained a broad zone of predominantly membranous material.

Five subcellular particulate fractions isolated during the A-XII zonal procedure were analysed to determine their hepatoma D23 specific antigenic activity. These fractions were as follows: (1) the total subcellular particulate preparation, sedimented at 78,000 *g* for 30 min from the whole homogenate; (2) the nuclear fraction sedimented from the whole homogenate at 1000 *g* for 12 min; (3) the extranuclear membrane fraction, sedimented at 78,000 *g* for 30 min from the

FIG. 1.—Fractionation of hepatoma D23 nuclear pellet suspension by sucrose density gradient centrifugation in an A-XII zonal rotor.

FIG. 2.—Absorption of anti-hepatoma D23 antibody by subcellular fractions isolated by A-XII zonal centrifugation. ■——■ total subcellular particulate preparation sedimented at 78,000 *g* for 30 min from the whole homogenate; ○——○ the nuclear fraction sedimented from the whole homogenate at 1000 *g* for 12 min; □——□ the extranuclear membrane fraction, sedimented at 78,000 *g* for 30 min from the 1000 *g* supernatant; ▲——▲ the material recovered from the sample zone of the A-XII zonal sucrose density gradient (tubes 3–10, Fig. 1); ●——● the membrane zone recovered from the A-XII zonal sucrose density gradient (tubes 26–36, Fig. 1).

1000 *g* supernatant; (4) the material recovered from the sample zone of the A-XII zonal sucrose density gradient (tubes 3–10, Fig. 1); (5) the membrane zone recovered from the A-XII zonal sucrose density gradient (tubes 26–36, Fig. 1).

The absorption of anti-hepatoma D23 antibody from tumour immune serum by these fractions, as assayed by inhibition of membrane immunofluorescence staining of hepatoma D23 cells, is shown in Fig. 2. Reduction of the fluorescence index (F.I.) to 0·3, this being taken to represent significant neutralization of specific antibody, required absorption with 31 mg protein/ml serum of the nuclear fraction. Since a greater amount (73 mg protein/ml) of the total subcellular particulate fraction was required for antibody absorption, initial centrifugation (1000 *g* for 12 min) increased the specific antigenic activity in the sedimented nuclear fraction whilst the extranuclear membrane (ENM) fraction (prepared from the 1000 *g* supernatant) was proportionately less antigenic. In this case, absorption with greater than 100 mg membrane protein was required to produce significant neutralization of specific antibody.

Two zones of subcellular material fractionated from the nuclear pellet suspension by A-XII zonal centrifugation exhibited higher antigenic activities Less than 8 mg/ml serum of protein from the broad zone containing membrane fragments (tubes 26–36) were required to produce significant neutralization of specific antibody. This represented an approximate ten-fold increase of hepatoma D23 specific antigenic activity compared with that associated with the total subcellular particulate preparation. The yield of protein from the region of the gradient containing the membrane zone (tubes 26–36) was within the range 0·5–1·5 mg/g of original hepatoma D23 tissue.

Although the A-XII zonal procedure allowed the isolation of a membrane fraction displaying higher tumour specific antigenic activity compared with that expressed in the unfractionated total subcellular particulate preparations, results were less satisfactory with respect to purity and homogeneity of the preparation. Much of the membranous material in this fraction sedimented through linear 30–60% (w/w) sucrose density gradients following centrifugation at 64,000 *g* for 15 h in swing-out rotors. Furthermore, phase contrast microscopic observations of the membrane fraction revealed the presence of some swollen and partially empty nuclei. Thus, this observation suggested that during homogenization and fractionation the stability of the hepatoma D23 nuclei was not fully preserved and probably the release of basic nuclear proteins which bind to subcellular membrane components (Wallach, 1967), occurred, preventing the isolation of a "clean" membrane fraction.

*B-XIV zonal centrifugation*

In view of the limitations of the A-XII zonal membrane preparation, a second preparative procedure was developed in which particular attention was paid to preserving nuclear integrity. The 0·25 mol/l sucrose homogenization medium and all sucrose solutions used in the B-XIV zonal fractionation contained 1 mmol/l $NaHCO_3$, 2 mmol/l $CaCl_2$, 2 mmol/l $MgCl_2$, pH 7·6, and at no stage during the preparation of the membrane fraction were nuclei exposed to a hypotonic environment or the absence of divalent cations.

The nuclear pellets prepared from hepatoma D23 homogenates by centrifugation at 1000 *g* for 30 min were dispersed in 60% (w/w) sucrose and introduced to the periphery of a B-XIV zonal rotor containing a discontinuous sucrose density gradient (see Materials and Methods). Figure 3 shows the OD 280 nm

FIG. 3.—Fractionation of hepatoma D23 nuclear pellet suspension by sucrose density gradient centrifugation in a B-XIV zonal rotor.

FIG. 4.—Electron micrograph of hepatoma D23 membranes prepared by B-XIV zonal centrifugation. Arrow indicates a contaminating mitochondrion. Thin sectioned. ×22,000.

and gradient profiles obtained upon unloading the rotor following centrifugation at 47,000 rev/min for 60 min. In a total of 10 individual membrane preparations from hepatoma D23, a defined zone of membranous material was resolved in 6–7 fractions (25 ml) located within tubes 6–12 (Fig. 3). This region of the gradient corresponded to the *d* 1·03/1·17 sucrose density discontinuity. The recovery of material in this membrane fraction was approximately 0·4 mg of protein/g of original hepatoma D23 tissue.

Examination of the material from the membrane zone of the gradient (tubes 6–12) by electron microscopy, using both negative contrast staining and thin sectioning techniques, revealed that this fraction consisted of smooth vesicular and fragmented membrane elements which were predominantly in small aggregates, (Fig. 4). The only morphologically identifiable contaminants of these preparations were mitochondria which were occasionally found localized at the centre of the larger membrane aggregates (Fig. 4). When the membrane fraction was sedimented by centrifugation (78,000 *g* for 30 min and resuspended in divalent cation-free medium (1 mmol/l $NaHCO_3$, pH 7·6) no aggregation of material occurred.

In one preparation of hepatoma D23 membranes, aliquots of the membrane fraction were subjected to isopycnic sucrose density gradient centrifugation. The membrane suspension recovered from the B-XIV zonal gradient (tubes 6–12) was diluted with 1/3 volume of 1 mmol/l $NaHCO_3$, 2 mmol/l $CaCl_2$, 2 mmol/l $MgCl_2$, pH 7·6, and sedimented by centrifugation at 78,000 *g* for 30 min. Pellets, each containing 9·5 mg of membrane protein, were resuspended in 2 ml of 18% (w/w) sucrose, layered upon 25 ml linear 20–45% (w/w) sucrose density gradients in the S.W. 25·1 swing-out rotor and centrifuged for 15 h at 64,000 *g*. When 1 mmol/l $NaHCO_3$, 2 mmol/l $CaCl_2$, 2 mmol/l $MgCl_2$, pH 7·6, was included in the 18% (w/w) sucrose resuspension medium and gradient solutions, a major diffuse band of membrane material was located at *d* 1·15–1·16. In the absence of divalent cations, however, the sucrose banding density of the membranes was determined to be *d* 1·14.

Four subcellular fractions, isolated during the B-XIV zonal procedure, were

FIG. 5.—Absorption of anti-hepatoma D23 antibody by subcellular fractions isolated by B-XIV zonal centrifugation. ■——■ total subcellular particulate preparation, sedimented from the whole homogenate at 78,000 *g* for 30 min; ○——○ the nuclear fraction sedimented from the whole homogenate at 1000 *g* for 30 min; □——□ the extranuclear membrane fraction, sedimented from the 1000 *g* supernatant by centrifugation at 78,000 *g* for 30 min; ●——● the membrane zone, isolated following centrifugation in the B-XIV zonal rotor.

examined for hepatoma D23 specific antigenic activity. These fractions were as follows: (1) the total subcellular particulate preparation, sedimented from the whole homogenate at 78,000 *g* for 30 min; (2) the nuclear fraction sedimented from the whole homogenate by centrifugation at 1,000 *g* for 30 min; (3) the extranuclear membrane fraction, sedimented from the 1000 *g* supernatant by centrifugation at 78,000 *g* for 30 min; (4) the membrane zone (tubes 6–12), isolated following centrifugation in the B-XIV zonal rotor.

Figure 5 illustrates the absorption of anti-hepatoma D23 antibody by these fractions, as determined by reduction of the membrane immunofluorescence staining of viable hepatoma D23 cells. Initial centrifugation of the whole homogenate at 1000 *g* resulted in the sedimentation of a fraction (nuclear fraction) which exhibited increased antigenic activity compared with the total subcellular particulate preparation. The membrane preparation obtained following B-XIV zonal centrifugation was, however, the most active antigenic fraction examined and only 8 mg of membrane protein/ml serum were required for significant neutralization of specific antibody.

*Hepatoma D23 nuclei*

In the two procedures adopted for the isolation of hepatoma D23 membranes, the most antigenic preparations were fractionated from nuclear sediments (1000 *g* pellets) of tumour homogenates. Studies were therefore undertaken to evaluate whether tumour specific antigen was expressed on hepatoma D23 nuclei. It was not possible to analyse directly for antigens on isolated nuclei using the membrane immunofluorescence test, since nonspecific uptake of the fluorescent antibody conjugate into the nucleoplasm prevented identification of any specific interactions. However, absorption of hepatoma D23 immune serum with nuclei ($5 \times 10^8$/ml serum) did not result in any demonstrable reduction of the serum F.I. (absorbed serum F.I., 0·63, unabsorbed serum F.I., 0·63). In comparison, pretreatment of serum with between $10^6$ and $10^7$ viable hepatoma D23 cells was sufficient to reduce the F.I. of immune serum to below 0·3, this being taken as the minimum value to represent a significant membrane immunofluorescence reaction. Furthermore, absorption of the standard hepatoma D23 immune serum (F.I., 0·72) with purified hepatoma D23 nuclear membranes (35 mg protein/ml serum) failed to effect significant neutralization of serum antibody (F.I., 0·53). These studies indicate that hepatoma D23 antigen is not demonstrable upon the nuclear membrane of the tumour cell.

### DISCUSSION

The present studies demonstrate that defined membrane preparations retaining hepatoma D23 specific antigenic activity may be isolated on a large scale from hepatoma homogenates by sucrose density gradient centrifugation in zonal rotors. These membrane fractions were obtained from the nuclear sediment either by rate dependent centrifugation in an A-XII zonal rotor or by flotation through sucrose solutions of *d* 1·17 in a B-XIV zonal rotor and, in both cases, the isolated preparations displayed an approximate ten-fold increase in hepatoma D23 specific antigenic activity compared with the total subcellular particulate fraction of homogenates. In contrast, no antigenic activity was found to be associated with purified hepatoma D23 nuclei or nuclear membranes.

The preparative procedure utilizing the B-XIV zonal rotor proved to be superior in its reproducibility and allowed the recovery of membrane fractions containing few morphologically identifiable contaminants. However, the two zonal procedures share a significant common feature in that essentially equivalent subcellular fractions isolated by both preparative methods exhibited the same order of antigenic activity, as defined by their capacity to absorb specific antibody from hepatoma D23 syngeneic immune serum. This order of antigenic activity of subcellular fractions was as follows: membranes taken from the zonal rotor > the nuclear sediment > total subcellular particulate fraction > extranuclear membrane fractions.

The methods developed for the isolation of antigenic membrane fractions are based primarily upon techniques previously used for the preparation of rat or mouse liver plasma membranes. Several authors have reported the use of the A-XII zonal rotor for rate dependent fractionation of the nuclear sediment of liver homogenates (reviewed by Hinton, 1972) and in liver fractionation studies, the 1 mmol/l $NaHCO_3$ (pH 7·6) homogenization medium, originally described by Neville (1960) for rat liver plasma membrane isolation, has been found to be satisfactory (Emmelot *et al.*, 1964; Song *et al.*, 1969; Evans, 1970). When applied to chemically induced rat hepatomata, however, nuclear disruption is promoted with the subsequent formation of a nucleoprotein gel, thus preventing the isolation of a plasma membrane fraction (Emmelot and Bos, 1966; Price *et al.*, 1972). The integrity of hepatoma D23 nuclei was found to be more adequately preserved with the addition of divalent cations to the homogenization media; nevertheless, the experiments using the A-XII zonal procedure emphasize the particular lability of hepatoma D23 nuclei in hypotonic media even though 2 mmol/l $Ca^{++}$ was included in the 1 mmol/l $NaHCO_3$ homogenization buffer. Although these studies showed that membranes of increased antigenic activity could be isolated by sucrose density gradient centrifugation in an A-XII zonal rotor, the final membrane material was contaminated with partially intact nuclear membranes identifiable by phase contrast microscopy.

The procedure developed for the preparation of antigenic hepatoma D23 membranes using a B-XIV zonal rotor was based upon the method reported by Touster *et al.* (1970) for the isolation of rat liver plasma membranes. With this latter technique, plasma membranes were prepared from either nuclear or microsomal fractions of homogenates after flotation through a discontinuous sucrose density gradient, and membranes were collected from an interface between a 37·2% (*i.e.* *d* 1·17) sucrose gradient solution and a 0·25 mol/l sucrose overlay solution. This method involved the use of a swing-out rotor which, compared with high speed zonal rotors, has a considerably lower loading capacity. Thus, by adapting the method of Touster *et al.* (1970) to incorporate fractionation in a zonal rotor, it was feasible to prepare hepatoma D23 membranes from the nuclear sediment from up to 100 g of tumour tissue. With these experiments, homogenization was performed in isotonic sucrose solutions containing low concentrations of $Ca^{++}$ and $Mg^{++}$ since this medium has previously been shown to be suitable for the preservation of hepatoma D23 nuclei (Price *et al.*, 1972). The membranes isolated using this method again exhibited an increased antigenic activity compared with other subcellular fractions obtained and the only identifiable contaminants to the preparations were mitochondria which were occasionally seen in the electron microscope at the centre of discrete membrane aggregates. The isopycnic banding density of the hepatoma D23 membranes in continuous sucrose density gradients after centrifugation in a swing-out rotor was 1·14 when experiments were performed in the absence of $Ca^{++}$ and $Mg^{++}$, this value being equivalent to that reported by Touster *et al.* (1970) for rat liver plasma membranes. However, a slightly higher density (*d* 1·15–1·16) was determined in the presence of 2 mmol/l $Ca^{++}$ and $Mg^{++}$ and this may be attributed to the fact that in this case the membranes sedimented in discrete aggregates with contaminating mitochondria these being of higher density (Anderson *et al.*, 1966). In support of this proposition, it was noted that no aggregates were observed in the electron microscope with membrane preparations suspended in dilute (1 mmol/l) bicarbonate solutions.

Two assumptions made in this investigation have been that the hepatoma D23 antigen is located primarily (if not exclusively) at the tumour cell plasma membrane and that methods reported for the isolation of liver plasma membranes would be applicable to hepatoma membranes. Whilst the methods adopted after modification were found to be suitable for the preparation of antigenic membrane fractions, it may not necessarily follow that all of the antigenic membrane elements were derived from the tumour cell plasma membrane. However, several observations suggest that the plasma membrane is a major subcellular location of hepatoma D23 specific antigen. Firstly, in order to elicit rejection reactions, these antigens must almost certainly show expression at the cell surface. Secondly, the indirect membrane immunofluorescence test has been employed in several studies to evaluate the unique specificity of the antigens associated with aminoazo dye induced rat hepatomata (Baldwin and Barker, 1967*b*; Baldwin *et al.*, 1971*a*, *b*). With this test, characteristic fluorescent ring reactions are observed to be localized to the surface of viable target cells, and also intact cells are capable of absorbing specific antibodies. Furthermore, in *in vitro* cytotoxicity tests or colony inhibition tests, hepatoma immune lymph node cells are capable of killing or modifying the growth characteristics of target tumour cells, this reaction almost certainly being dependent upon the establishment of cell contact (Baldwin and Embleton, 1971; Baldwin *et al.*, 1973*b*). Also, the finding that tumour specific antigens may be solubilized by enzymic treatment of intact cells indicates a surface localization for these antigens (Harris *et al.*, 1973).

Similar evidence has been put forward to support the proposition that transplantation antigens are located at the surface membrane of mammalian cells and several studies have attempted to correlate the antigenic activity of subcellular fractions with enzymic markers which are considered to be characteristic of the plasma membrane and other subcellular organelles. It may be concluded from these investigations that while H-2 antigens may be expressed to some extent on internal membrane elements (including the nuclear membrane, Albert and Davies, 1973), there is often a high enrichment of these antigens in the surface or plasma membrane fractions (Herberman and Stetson, 1965; Haughton, 1966; Ozer and Wallach, 1967; Molnar, Klein and Friberg, 1973). The inference from the present studies is that the hepatoma D23 specific antigen may share a similar expression and subcellular localization.

This study was supported by the Cancer Research Campaign and by a Government Equipment Grant through the Royal Society. The authors wish to thank Dr J. R. Harris, Department of Physiology, Bute Medical Buildings, St Andrews, Fife for performing thin sectioning and negative staining electron microscopic analysis of subcellular fractions.

### REFERENCES

ALBERT, W. H. W. & DAVIES, D. A. L. (1973) H-2 Antigens on Nuclear Membranes. *Immunology*, **24**, 841.

ANDERSON, N. G., HARRIS, W. W., BARBER, A. A., RANKIN, C. T. & CANDLER, E. L. (1966) Separation of Subcellular Components and Viruses by Combined Rate- and Isopycnic-Zonal Centrifugation. *Natn. Cancer Inst. Monog.*, **21**, 253.

BALDWIN, R. W. & BARKER, C. R. (1967*a*) Tumour specific Antigenicity of Aminoazo-dye-induced Rat Hepatomas. *Int. J. Cancer*, **2**, 355.

BALDWIN, R. W. & BARKER, C. R. (1967*b*) Demonstration of Tumour-specific Humoral Antibody against Aminoazo Dye-induced Rat Hepatomata. *Br. J. Cancer*, **21**, 793.

BALDWIN, R. W. & EMBLETON, M. J. (1971) Demonstration by Colony Inhibition Methods of Cellular and Humoral Immune Reactions to Tumour-specific Antigens Associated with Aminoazo-dye-induced Rat Hepatomas. *Int. J. Cancer*, **7**, 17.

BALDWIN, R. W. & GLAVES, D. (1972) Solubilization of Tumour-specific Antigen from Plasma Membrane of an Aminoazo-dye-induced Rat Hepatoma. *Clin. & exp. Immunol.*, **11**, 51.

BALDWIN, R. W. & MOORE, M. (1969) Isolation of Membrane-associated Tumour-specific Antigen from an Aminoazo-dye-induced Rat Hepatoma. *Int. J. Cancer*, **4**, 753.

BALDWIN, R. W., BARKER, C. R., EMBLETON, M. J., GLAVES, D., MOORE, M. & PIMM, M. V. (1971*a*) Demonstration of Cell-surface Antigens on Chemically-induced Tumors. *Ann. N.Y. Acad. Sci.*, **177**, 268.

BALDWIN, R. W., GLAVES, D. & PIMM, M. V. (1971*b*) Tumor-associated Antigens as Expressions of Chemically-induced Neoplasia and their Involvement in Tumor-Host Interactions. In *Progress in Immunology*. Ed. B. Amos. New York: Academic Press. p. 907.

BALDWIN, R. W., EMBLETON, M. J. & MOORE, M. (1973*a*) Immunogenicity of Rat Hepatoma Membrane Fractions. *Br. J. Cancer*, **28**, 389.

BALDWIN, R. W., EMBLETON, M. J. & ROBINS, R. A. (1973*b*) Cellular and Humoral Immunity to Rat Hepatoma-specific Antigens Correlated with Tumour Status. *Int. J. Cancer*, **11**, 1.

BALDWIN, R. W., HARRIS, J. R. & PRICE, M. R. (1973*c*) Fractionation of Plasma Membrane-associated Tumour Specific Antigen from an Aminoazo Dye-induced Rat Hepatoma. *Int. J. Cancer*, **11**, 385.

BALDWIN, R. W., PRICE, M. R. & ROBINS, R. A. (1973*d*) Inhibition of Hepatoma Immune Lymph Node Cell Cytotoxicity by Tumour Bearer Serum and Solubilized Hepatoma Antigen. *Int. J. Cancer*, **11**, 527.

EMMELOT, P. & BOS, C. J. (1966) Differences in the Association of Two Glycolytic Enzymes with Plasma Membranes Isolated from Rat Liver and Hepatoma. *Biochim. biophys. Acta.*, **121**, 434.

EMMELOT, P., BOS, C. J., BENEDETTI, E. L. & RÜMKE, PH (1964) Studies on Plasma Membranes. I. Chemical Composition and Enzyme Content of Plasma Membrane Isolated from Rat Liver. *Biochim. biophys. Acta*, **90**, 126.

EVANS, W. H. (1970) Fractionation of Liver Plasma Membranes Prepared by Zonal Centrifugation. *Biochem. J.*, **166**, 833.

HARRIS, J. R., PRICE, M. R. & BALDWIN, R. W. (1973) The Purification of Membrane-associated Tumour Antigens by Preparative Polyacrylamide Gel Electrophoresis. *Biochim. biophys. Acta*, **311**, 600.

HAUGHTON, G. (1966) Transplantation Antigen of Mice. Cellular Location of Antigen Determined by the H-2 Locus. *Transplantation*, **4**, 238.

HERBERMAN, R. & STETSON, C. A. (1965) The Expression of Histocompatibility Antigens on Cellular and Subcellular Membranes. *J. exp. Med.*, **121**, 533.

HINTON, R. H. (1972) Purification of Plasma-membrane Fragments. In *Subcellular Components: Preparation and Fractionation*. Ed. G. D. Birnie, 2nd Edn. London: Butterworths. p. 119.

LOWRY, O. H., ROSEBROUGH, N. J., FARR, A. L. & RANDALL, R. J. (1951) Protein Measurement with the Folin Phenol Reagent. *J. biol. Chem.*, **193**. 265.

MOLNAR, J., KLEIN, G. & FRIBERG, S. (1973)

Subcellular Localization of Murine Histocompatibility Antigens in Tumor Cells. *Transplantation*, **16**, 93.

NEVILLE, D. M. (1960) The Isolation of a Cell Membrane Fraction from Rat Liver. *J. biophys. biochem. Cytol.*, **8**, 413.

OETTGEN, H. F., OLD, L. J., MCLEAN, E. P. & CARSWELL, E. A. (1968) Delayed Hypersensitivity and Transplantation Immunity Elicited by Soluble Antigens of Chemically Induced Tumours in Inbred Guinea Pigs. *Nature, Lond.*, **220**, 295.

OZER, J. H. & WALLACH, D. F. H. (1967) H-2 Components and Cellular Membranes. Distinction between Plasma Membrane and Endoplasmic Reticulum Governed by the H-2 Region in the Mouse. *Transplantation*, **5**, 562.

PRICE, M. R. & BALDWIN, R. W. (1974) Immunogenic Properties of Rat Hepatoma Subcellular Fractions. *Br. J. Cancer*, **30**, 394.

PRICE, M. R., HARRIS, J. R. & BALDWIN, R. W. (1972) A Method for the Isolation and Purification of Normal Rat Liver and Hepatoma Nuclear "Ghosts" by Zonal Centrifugation. *J. ultrastruct. Res.*, **40**, 178.

SONG, C. S., RUBIN, W., RIFKIND, A. B. & KAPPAS, A. (1969) Plasma Membranes of Rat Liver. Isolation and Enzymatic Characterization of a Fraction Rich in Bile Canaliculi. *J. cell Biol.*, **41**, 124.

SUTER, L., BLOOM, B. R., WADSWORTH, E. M. & OETTGEN, H. F. (1972) Use of the Macrophage Migration Inhibition Test to Monitor Fractionation of Soluble Antigens of Chemically Induced Sarcomas of Inbred Guinea Pigs. *J. Immun.*, **109**, 766.

THOMSON, D. M. P. & ALEXANDER, P. (1973) A Cross-reacting Embryonic Antigen in the Membrane of Rat Sarcoma Cells which is Immunogenic in the Syngeneic Host. *Br. J. Cancer*, **27**, 35.

TOUSTER, O., ARONSON, N. N., DULANEY, J. T. & HENDRICKSON, H. (1970) Isolation of Rat Liver Plasma Membranes. Use of Phosphodiesterase I as Marker Enzymes. *J. cell Biol.*, **47**, 604.

WALLACH, D. F. H. (1967) Isolation of Plasma Membranes of Animals Cells. In *The Specificity of Cell Surfaces*. Ed. B. D. Davis and L. Warren. Eaglewood Cliffs, N.J.: Prentice-Hall, Inc. p. 129.